# Extensive colonic pneumatosis in a patient on adjuvant chemotherapy after right colectomy for primary terminal ileum lymphoma: A decision-making process between surgical and non-surgical management

**DOI:** 10.1016/j.ijscr.2018.09.050

**Published:** 2018-10-09

**Authors:** M. Kirmanidis, K.A. Boulas, A. Paraskeva, I. Kariotis, N. Barettas, S. Kariotis, Ch. Keskinis, A. Hatzigeorgiadis

**Affiliations:** Department of General Surgery, General Hospital of Drama, Drama, Greece

**Keywords:** Pneumatosis intestinalis, Pneumatosis coli, Chemotherapy, Primary intestinal lymphoma, Case report

## Abstract

**Introduction:**

Pneumatosis intestinalis is a rare condition that may be idiopathic or a sign of numerous underlying gastrointestinal, pulmonary and systemic diseases.

**Presentation of case:**

Herein, the case an otherwise-healthy 82-year-old female patient with vague abdominal pain due to total colonic pneumatosis 20 days after completion of R-CHOP chemotherapy for a stage IIE primary non-Hodgkin’s lymphoma of the terminal ileum submitted to right hemicolectomy and ileal resection 6 months previously is presented. As no evidence of intramural bowel gas was present on pre-operative CT, pneumatosis coli considered to be secondary. As no worrisome clinical, laboratory and imaging findings were present, pneumatosis coli seemed to be benign. As no other etiologic factors identified, pneumatosis coli considered to be chemotherapy-induced. The patient treated conservatively with cessation of enteral nutrition and broad spectrum antibiotics with uneventfull recovery.

**Discussion:**

Pneumatosis intestinalis can be benign or life-threatening. Bowel obstruction, perforation, ischemia and severe colitis represent the most life-threatening causes. In clinical practice it is often challenging to distinguish between life-threatening and benign pneumatosis intestinalis, a decision which should be based on the presence or absence of worrisome clinical, laboratory and imaging findings.

**Conclusion:**

In analogous cases, the main dilemma for the physicians is to identify whether surgical intervention is required or not. Given the potential severity of pneumatosis intestinalis, early diagnosis and recognition of its severity is critical as it would dictate surgical or non-surgical management.

## Highlights

• Pneumatosis intestinalis can be benign or life-threatening.
• Life-threatening causes are bowel obstruction, perforation, ischemia and severe colitis.
• Differential diagnosis between life-threatening and benign pneumatosis intestinalis is difficult.
• Early severity recognition is critical as it would dictate surgical or conservative management.
• Surgery is needed when worrisome clinical, laboratory and imaging findings are present.

## 1 Introduction

*This work has been reported in line with the SCARE criteria* [1].

Pneumatosis intestinalis (PI) is an imaging phenomenon representing the presence of gas in the bowel wall. Based on autopsy studies, its incidence has been estimated as 3 per 10,000 individuals in the general population. Intramural gas may originate from intraluminal gastrointestinal gas, bacterial gas production, pulmonary gas, and its collection in the bowel wall may be explained by the following pathophysiological mechanisms: (a) bowel necrosis; (b) mucosal disruption; (c) increased mucosal permeability; and (d) pulmonary disease [2]. Bowel obstruction, perforation, ischemia and severe colitis represent the most life-threatening causes of PI. However, PI may be the result of numerous non-ischemic and non-obstructive gastrointestinal conditions along with various pulmonary and systemic conditions (Table 1) [3]. In clinical practice, it is often challenging for the physician to distinguish between benign and life-threatening PI, a decision which would dictate non-surgical and surgical management, respectively.

**Table 1:** Causes and pathophysiology of pneumatosis intestinalis (PI).

Herein, the case an otherwise-healthy 82-year-old female patient with vague abdominal pain due to total colonic pneumatosis 20 days after completion of R-CHOP chemotherapy for a stage IIE primary non-Hodgkin’s lymphoma of the terminal ileum submitted to right hemicolectomy and ileal resection 6 months previously is presented. The question whether pneumatosis coli represented a surgical emergency inevitably arises. The present case report is educational as it describes the dynamic decision making process for differential diagnosis between surgical and non-surgical PI, and unique due to the unusual presentation with the presence of intramural air collection throughout the entire remaining colonic wall without any predominate symptoms and signs.

## 2 Presentation of case

**Fig. 1:**
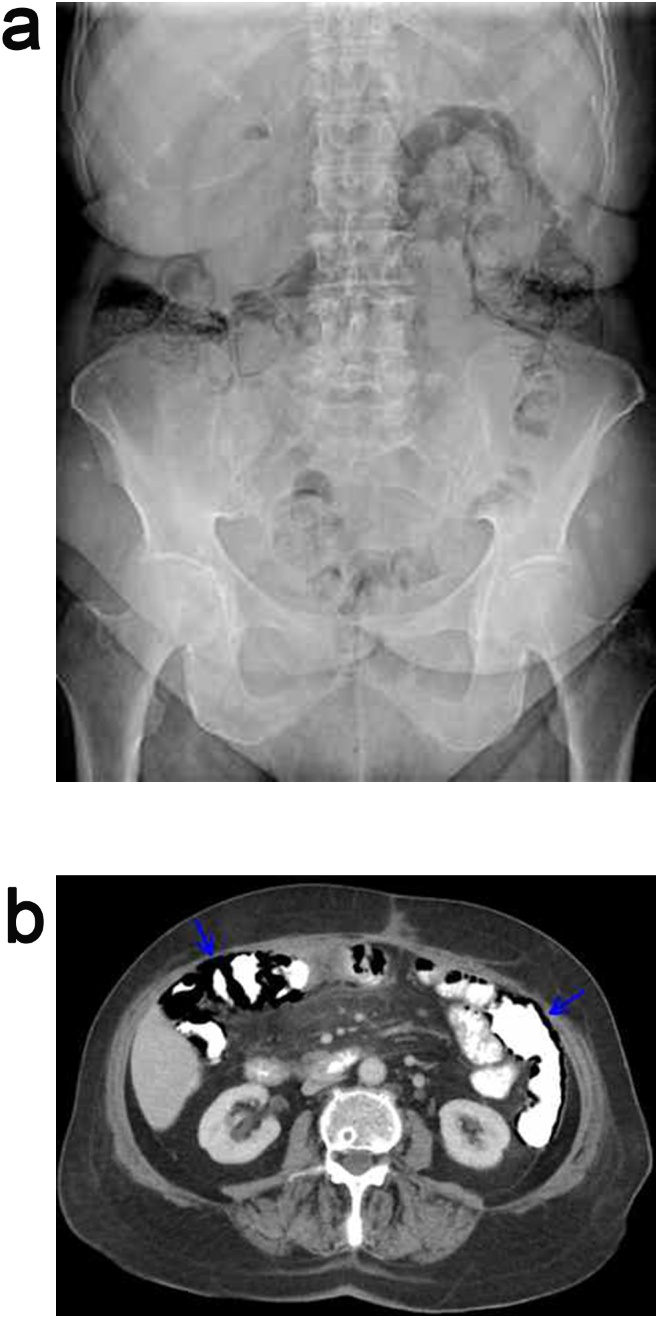
Abdominal (a) radiograph and (b) CT at initial presentation showing total linear submucosal and subserosal pneumatosis of the remaining colon with free intraperitoneal air.

An otherwise-healthy 82-year-old female patient, with a history of right hemicolectomy and ileal resection 6 months previously for a low intermediate risk, stage IIE, primary diffuse large B-cell non-Hodgkin’s lymphoma of the terminal ileum, presented to the emergency department complaining of vague, constant, diffuse abdominal pain with no concurrent symptoms over the preceding 3 days [4]. The patient had completed 8 cycles of chemotherapy 20 days ago with the R-CHOP regimen (cyclophosphamide 750 mg/m^2^, doxorubicin 50 mg/m^2^, vincristine 1.4 mg/m^2^ and rituximab 375 mg/m^2^ by intravenous infusion on day 1, oral prednisolone 40 mg/m^2^ on days 1–5 administered every 21 days) [5]. At initial presentation, vital signs were within the normal range, physical examination of the abdomen was normal, WBC count, CRP levels were normal, and arterial blood gas analysis was within normal reference range. Abdominal radiograph (Fig. 1a) and CT (Fig. 1b) showed linear submucosal and subserosal intramural gas collection along the wall of the entire remaining colon with free intraperitoneal air and without evidence of acute mesenteric arterial occlusion, portal venous gas, bowel dilatation and ascites. Stool cultures were negative for enteric pathogens.

Based on the present imaging findings, the patient diagnosed with total colonic pneumatosis. As no evidence of intramural bowel gas was present on pre-operative staging CT, pneumatosis coli considered to be secondary. Based on initial assessment, life-threatening etiological factors such as bowel obstruction, perforation, ischemia and severe colitis were excluded; secondary survey with repeated clinical, laboratory and imaging evaluation initiated. On re-assessment, pneumatosis coli seemed to be benign as no worrisome clinical and laboratory findings such as hypotension, tachycardia, acute abdomen on physical exam, metabolic acidosis, elevated WBC count and acute renal failure developed, and no worrisome imaging findings such as ascites, portal venous gas and bowel dilatation on CT developed. The patient treated conservatively with cessation of enteral nutrition and broad spectrum antibiotics (2^nd^ generation cephalosporin and metronidazole). After a one week hospital stay, interval abdominal radiograph and CT showed almost complete resolution of imaging findings; the patient referred to a tertiary hospital for hematology and oncology specialty care.

## 3 Discussion

The present report describes a patient with a stage IIE non-Hodgkin’s lymphoma of the terminal ileum who developed benign secondary total colonic pneumatosis after receiving 8 cycles of R-CHOP adjuvant chemotherapy which treated conservatively. In analogous cases, the main dilemma for the physician is to identify whether surgical intervention is required or not. The decision making process (Table 2) for distinguishing surgical and non-surgical PI is presented below.

**Table 2:** Decision making flow-chart for patients diagnosed with pneumatosis intestinalis (PI).

### 3.1 Step 1-primary or secondary pneumatosis intestinalis?

PI can be divided into primary and secondary which attribute to 15% and 85% of cases, respectively. Primary PI is a benign condition, usually asymptomatic and incidentally discovered on imaging. Secondary PI may be the result of numerous life-threatening, potential life-threatening and benign gastrointestinal conditions along with various pulmonary and systemic conditions, as shown in Table 1 [6]. Secondary PI is usually symptomatic; the most common symptoms include abdominal pain (53.9%), followed by diarrhea (53.0%), abdominal distention (42.4%), nausea and vomiting (14.3%), bloody and mucous stool *(12.9% and* 12.0%, respectively) and constipation (7.8%) [7].

In the present case, the patient complained of vague abdominal pain. The patient had a history of right hemicolectomy and ileal resection 6 months previously for a stage IIE primary non-Hodgkin’s lymphoma of the terminal ileum submitted to 8 cycles of R-CHOP chemotherapy. Abdominal CT at initial presentation revealed the presence of total colonic pneumatosis which considered being secondary as no evidence of intramural bowel gas was present on pre-operative staging CT. The question that arised immediately was what was the cause. However on primary survey, it is emerging for the physician not to thoroughly delineate etiology but to distinguish between benign or worrisome secondary PI; ascertaining whether a life-threatening cause such as bowel obstruction, perforation, ischemia and severe colitis exist or not based on clinical, laboratory and imaging evaluation is the primary priority.

### 3.2 Step 2- benign or worrisome secondary pneumatosis intestinalis? Clinical and laboratory evaluation

In 2013, the Eastern Association for the Surgery of Trauma, in a retrospective multicenter study of 500 patients with PI, showed that lactate value of more than 2.0 mmol/L, hypotension, peritonitis, acute renal failure, active mechanical ventilation and absent bowel sounds were significantly correlated with worrisome PI. The authors concluded that the presence of a lactate value of more than 2.0 mmol/L and hypotension had a predictive probability for life-threatening PI of 93.2% [8]. In 2017, the American Association for the Surgery of Trauma, in a prospective multicenter study of 127 patients with PI, showed that clinical findings of ileus, peritoneal signs on physical examination, sepsis, hypotension and laboratory findings of elevated INR, decreased hemoglobin, lactate value of more than 2.0 mmol/L were significantly correlated with worrisome PI. The authors concluded that surgical exploration should be strongly recommended for patients with PI presenting with a lactate value greater than 2 mmol/L and peritonitis [9]. In the present case, no worrisome clinical and laboratory findings were present on primary and secondary surveys; consequently pneumatosis coli seemed to be benign.

### 3.3 Step 3- benign or worrisome secondary pneumatosis intestinalis? Imaging evaluation

In 2017, Goyal et al, in a retrospective study of 167 patients with PI, showed that location in the small bowel, bowel dilation, mesenteric stranding, bowel enhancement, portal vein gas, mesenteric vein gas and moderate mesenteric edema were imaging findings significantly correlated with worrisome PI [10]. In 2013, Lee et al., in a retrospective study of 84 patients with PI, showed that bowel wall thickening, mesenteric stranding, ascites, bowel dilation, location in the small bowel, portal vein gas and mesenteric vein gas were imaging findings significantly correlated with worrisome PI [11]. In the present case, no worrisome imaging findings were present on primary and secondary survey; consequently pneumatosis coli seemed to be benign.

### 3.4 Step 4-identifying etiology

On primary survey it is of primary priority for the physician to ascertain whether a life-threatening cause of PI such as bowel obstruction, perforation, ischemia and severe colitis exist or not based on the presence of worrisome clinical, laboratory and imaging findings. The above surgical emergency situations should always kept in mind during secondary surveys and once excluded further delineation of the exact etiology should be initiated (Table 1). In the present case, pneumatosis coli seemed to be benign as no worrisome clinical, laboratory and imaging findings were present during re-assessments. As no other etiologic factors identified, pneumatosis coli considered to be chemotherapy-induced.

Chemotherapy agents, such as fluorouracil and docetaxel, and molecular targeting agents, such as bevacizumab and sunitinib, may rarely cause PI. The underlying mechanisms are thought to be increased mucosal permeability and decreased submucosal lymphoid tissue [12]. Although PI in patients on chemotherapy is usually a benign and totally reversible situation after cessation of therapy, it can also be life-threatening when it is the result of chemotherapy-induced ischemic enteritis and colitis associated with chemotherapeutic agents such as docetaxel and bevacizumab [13].

### 3.5 Step 5-determining management: surgical or non-surgical treatment?

PI patients with worrisome clinical, laboratory and imaging findings should undergo surgical treatment. In the absence of worrisome findings, patients should undergo a trial of non-surgical treatment under strict re-evaluation. Non-surgical management should focus on treating the underlying gastrointestinal or systemic cause and discontinuation of possible instigating medications. Antibiotics, especially metronidazole 500 mg per os three times daily for up to 3 months or until documented resolution of PI, can be used to target intraluminal and intramural bacteria in order to reduce anaerobic production of hydrogen gas [14]. Cessation of oral diet and introduction of an elemental diet, which is totally absorbed in the small intestine, seems to be useful by decreasing production of gas by colonic flora. However, elemental diet is not always well tolerated and bowel hypomotility may increase intraluminal pressure and worsen the PI. Accordingly, case reports documenting investigative therapy with promotility agents such as metoclopramide and erythromycin exist in the literature, though data are limited [15].

Oxygen therapy has long been recognized as an effective therapy for PI leading to cyst regression on imaging and symptoms resolution by the following mechanisms: (a) increased tissue oxygenation may facilitate phagocytic activity and directly target the gas-producing organisms via anaerobic impairment; and (b) increased arterial oxygen tension forces oxygen into the hydrogen-containing cysts by diffusion from areas of high oxygen tension in the artery to low oxygen tension in the cysts. In turn, oxygen accumulation in the cysts increases the partial pressure of hydrogen in the cysts causing hydrogen to diffuse out of the high-pressure cyst into the low-hydrogen bloodstream. Cyst resolution follows as the oxygen leaves the cyst via re-absorption for use in cellular metabolism. A trial of hyperbaric oxygen therapy can be used at 2.5 ATA for 2.5 h for at least 3 sessions or until 2 days after the disappearance of cysts to reduce the risk of recurrence [16].

## 4 Conclusions

PI is a rare condition that may be idiopathic or a sign of an underlying gastrointestinal or systemic disease. Given the potential severity of this condition, early diagnosis and recognition of the severity by evaluating certain worrisome clinical, laboratory and imaging findings is critical. Treatment includes surgery or non-surgical treatment by bowel rest, cessation of instigating medications, antibiotic therapy and oxygen therapy.

## Conflicts of interest

None.

## Funding

None.

## Ethical approval

Ethical approval has been exempted by our institution.

## Consent

“Written informed consent was obtained from the patient for publication of this case report and accompanying images. A copy of the written consent is available for review by the Editor-in-Chief of this journal on request”.

## Author contributions

Boulas K was responsible for the study concept and design. Paraskeva A, Kariotis I, Kariotis S, Keskinis Ch and Barettas N equally contributed in writing the paper. Blouhos K and Hatzigeorgiadis A had the final approval of the paper.

## Registration of research studies

No unique identifying number requested for this case report.

## Guarantor

Hatzigeorgiadis A.

## Peer review and provenance

Not commissioned, externally peer reviewed.
